# Enhancer LncRNAs Influence Chromatin Interactions in Different Ways

**DOI:** 10.3389/fgene.2019.00936

**Published:** 2019-10-16

**Authors:** Yue Hou, Rongxin Zhang, Xiao Sun

**Affiliations:** State Key Laboratory of Bioelectronics, School of Biological Science and Medical Engineering, Southeast University, Nanjing, China

**Keywords:** chromatin structure, enhancer lncRNA, enhancer–promoter interaction, Hi-C, transcription factor

## Abstract

More than 98% of the human genome does not encode proteins, and the vast majority of the noncoding regions have not been well studied. Some of these regions contain enhancers and functional non-coding RNAs. Previous research suggested that enhancer transcripts could be potent independent indicators of enhancer activity, and some enhancer lncRNAs (elncRNAs) have been proven to play critical roles in gene regulation. Here, we identified enhancer–promoter interactions from high-throughput chromosome conformation capture (Hi-C) data. We found that elncRNAs were highly enriched surrounding chromatin loop anchors. Additionally, the interaction frequency of elncRNA-associated enhancer–promoter pairs was significantly higher than the interaction frequency of other enhancer–promoter pairs, suggesting that elncRNAs may reinforce the interactions between enhancers and promoters. We also found that elncRNA expression levels were positively correlated with the interaction frequency of enhancer–promoter pairs. The promoters interacting with elncRNA-associated enhancers were rich in RNA polymerase II and YY1 transcription factor binding sites. We clustered enhancer–promoter pairs into different groups to reflect the different ways in which elncRNAs could influence enhancer–promoter pairs. Interestingly, G-quadruplexes were found to potentially mediate some enhancer–promoter interaction pairs, and the interaction frequency of these pairs was significantly higher than that of other enhancer–promoter pairs. We also found that the G-quadruplexes on enhancers were highly related to the expression of elncRNAs. G-quadruplexes located in the promoters of elncRNAs led to high expression of elncRNAs, whereas G-quadruplexes located in the gene bodies of elncRNAs generally resulted in low expression of elncRNAs.

## Introduction

It has been widely accepted that a large proportion of the human genome is transcribed, but that less than 2% of the transcripts are subsequently translated into proteins (Katayama et al., 2005; Djebali et al., 2012; Sallam et al., 2018). Long non-coding RNAs (lncRNAs), transcripts longer than 200 nucleotides, have attracted increasing attention because of their functional relevance in various biological processes (Iyer et al., 2015; Liu et al., 2017; Kopp and Mendell, 2018). Because lncRNAs are expressed at relatively low levels and are weakly conserved during evolution, they are difficult to annotate and were historically regarded as junk DNA (Uszczynska-Ratajczak et al., 2018). However, convincing evidence has recently emerged that at least some lncRNAs play critical roles in disease (Wapinski and Chang, 2011; Shi et al., 2013; Yan et al., 2015; Wan et al., 2016; Zhang et al., 2017), organism development (Grote et al., 2013; Fatica and Bozzoni, 2014; Sun et al., 2017), and aging (Bianchessi et al., 2015; Yang et al., 2016a; Neppl et al., 2017). Iyer et al. identified 58,648 lncRNA genes in the human genome, of which 1% harbored ultraconserved elements and 7% overlapped with disease-associated SNPs (Iyer et al., 2015). Using a CRISPR interference platform, hundreds of lncRNAs were proven to be required for robust cellular growth for different cell types (Liu et al., 2017). Some lncRNAs can regulate the expression of neighboring (*cis*) or distal (*trans*) genes (Yu et al., 2018). *In cis* means that lncRNAs regulate target genes by the act of transcription (Tehrani et al., 2018). LncRNAs, such as *bxd* lncRNA (Hao et al., 2017), can regulate downstream promoters *in cis* through transcriptional interference (Lin et al., 2018). In addition to acting *in cis*, some lncRNAs translocate from their sites of synthesis and regulate distal target genes *in trans* (Kopp and Mendell, 2018). For instance, *Firre* lncRNA localizes at five distinct *trans*-chromosomal loci through interacting with the nuclear-matrix factor hnRNPU (Hacisuleyman et al., 2014; Yang et al., 2015). Moreover, the *Xist* lncRNA participates in silencing transcription *in trans* by interacting with SHARP (McHugh et al., 2015).

Based on their genomic organization, lncRNAs can be categorized into different subtypes, including intragenic lncRNAs, intergenic lncRNAs, and enhancer lncRNAs (elncRNAs) (Devaux et al., 2015; St Laurent et al., 2015). Enhancers are genomic regions that are bound by transcription factors (TFs) and are capable of interacting with promoters to augment gene expression. Generally, enhancer regions are marked by histone 3 lysine 4 monomethylation (H3K4me1) and histone 3 lysine 27 acetylation (H3K27ac). The binding of the general transcriptional co-activator CBP to enhancers may recruit RNA polymerase II (RNA POLII) and produce enhancer transcripts (Kim et al., 2010). Pioneering research has proven that enhancer RNAs are involved in specific enhancer–promoter looping initiated by ER-α binding (Li et al., 2013). In addition to affecting enhancer–promoter loops, some elncRNAs regulate gene expression by recruiting TFs to the promoters of target genes. LEENE, an elncRNA that enhances eNOS expression, can facilitate the recruitment of RNA POLII to the eNOS promoter to enhance eNOS nascent RNA transcription (Miao et al., 2018). Arc eRNA, an elncRNA that is expressed from the enhancer for Activity-regulated cytoskeletal protein (Arc), can facilitate NELF release from the target promoter (Schaukowitch et al., 2014). Moreover, a muscle-specific elncRNA, DRReRNA, regulates the transcription of myogenin in *trans* by mediating the recruitment of cohesin proteins (Tsai et al., 2018). In principle, nascent RNAs can remain at their sites of synthesis. One of the well-studied mechanisms for retaining nascent RNA is through the formation of an R-loop, which is a double-stranded RNA:DNA hybrid opposite a displaced single strand of DNA (Li and Fu, 2019). R-loops, which are associated with transcription activities under physiological conditions (Skourti-Stathaki et al., 2011; Stork et al., 2016), predominantly form on promoters and enhancers associated with GC-skewed sequences (Ginno et al., 2012; Chen et al., 2017; Li and Fu 2019). These findings suggested that elncRNAs might stay where they are synthesized but exert long-distance regulatory effects on target genes.

Previous studies provided great advances in our understanding of the functions of elncRNAs. However, some studies roughly coupled enhancers to their closest genes, which has been proven to be an imprecise method for identifying the target genes of enhancers. DNA is highly compacted in the nucleus, resulting in a complicated three-dimensional genome conformation. Currently, the developed powerful Hi-C technology has been used to profile the three-dimensional chromatin structure in diverse organisms and cells (Lieberman-Aiden et al., 2009; Rao et al., 2014; Mifsud et al., 2015). As enhancers and their target promoters frequently contact each other despite being separated by thousands or millions of base pairs in genomic distance (Ay et al., 2014), several methods have been proposed to identify enhancers and their target genes using Hi-C (Whalen et al., 2016; Ron et al., 2017). Mifsud et al. proposed that transcriptionally active genes normally interact with regulatory elements and inactive genes frequently interact with genomic regions that are rich in repressive markers (Mifsud et al., 2015). Beagrie et al. found an abundance of three-way contacts among highly transcribed regions (Beagrie et al., 2017). Moreover, specific enhancer transcripts have been proven to be involved in maintaining the formation of loop structures (Lai et al., 2013; Li et al., 2013; Hsieh et al., 2014; Yang et al., 2016b). However, it remains a challenge to decipher the function and mechanism of elncRNAs in the genome-wide range.

In this study, we comprehensively characterized elncRNAs by analyzing the human chromatin structure. Using Hi-C data, chromatin loops and enhancer–promoter interactions were identified in the GM12878 cell line. Our study was intended to resolve the following issues: 1) whether chromatin loops are associated with elncRNAs in the genome-wide range; 2) whether enhancer–promoter interactions are influenced by elncRNAs in the genome-wide range; and 3) the relationship between elncRNAs and transcription factor binding sites (TFBSs). We found that chromatin loops and enhancer–promoter interactions were highly associated with elncRNAs. By analyzing the relationship between elncRNAs and TFBSs, we found that elncRNAs are capable of affecting TFBSs on both local enhancers and target promoters. Our findings suggest that elncRNAs influence enhancer–promoter interactions in different ways.

## Materials and Methods

### Identification of Genomic Elements

The protein-coding and lncRNA genes in the human genome were downloaded from the GENCODE (Harrow et al., 2012) and NONCODE (Fang et al., 2018) databases, respectively. A total of 19,901 protein-coding genes and 96,308 lncRNA genes were identified. In accordance with previous research (He et al., 2014), promoters were defined as regions located 2 kilo-base pairs (kb) upstream and 0.5 kb downstream of transcription start sites (TSSs) annotated in GENCODE (Harrow et al., 2012).

Genomic regions of enhancers in the GM12878 cell line were derived from a previous study (Yip et al., 2012). Enhancers located in promoters and gene bodies of protein-coding genes were excluded. After filtering, a total of 35,939 enhancers in the GM12878 cell line were retained.

### Global Nuclear Run-On Sequencing Data and RNA Sequencing Data

The global nuclear run-on sequencing (GRO-seq) data of the GM12878 cell line were generated by Core et al. (GEO accession number: GSE60456) (Core et al., 2014). GRO-seq captures 5′-capped RNAs from active transcriptional regulatory elements with high accuracy (Danko et al., 2015). The obtained GRO-seq reads were mapped to the human reference genome (GRCh37/hg19) using *Bowtie2* (Langmead and Salzberg, 2012). We used dREG, a computational tool for identifying transcriptional regulatory DNA sequences using GRO-seq data, to call peaks (Danko et al., 2015).

The paired-end RNA-seq data of the GM12878 cell line (GEO accession number: GSE90223) were generated by Thomas Gingeras’ group of the ENCODE Consortium (Consortium, 2012). RNA-seq reads were mapped to the human reference genome (GRCh37/hg19) by *tophat* (Trapnell et al., 2012). We used *cufflinks* to generate the transcriptome assembly (Trapnell et al., 2010) and *cuffdiff* to test for differential expression (false discovery rate (FDR) <0.05; fold change >1.5) (Trapnell et al., 2013). As reported previously, numerous lncRNAs are expressed at much lower levels than protein-coding genes (Derrien et al., 2012); therefore, we used a threshold of 0.21 fragments per kilobase of transcript per million fragments mapped (FPKM) to define expressed lncRNAs, in accordance with previous studies (Hart et al., 2013; Bonnal et al., 2015).

### Identification of elncRNAs

**Figure 1:**
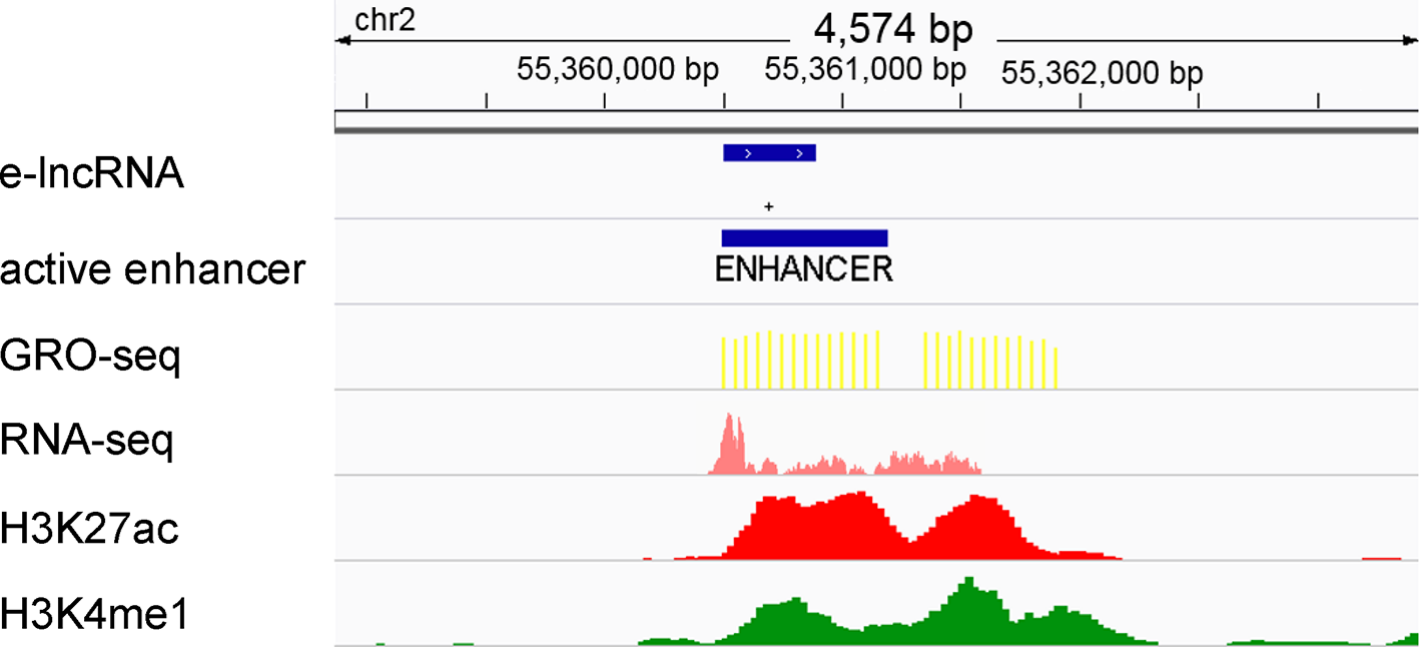
Definition of active enhancers and elncRNAs. Enhancers were predicted by ChromHMM and Segway according to the histone modifications surrounding them. The enhancers that fall within 1 kb of the GRO-seq peaks were defined as active enhancers. LncRNA genes overlapping with active enhancers were defined as elncRNAs.

It has been proven that active transcriptional regulatory elements can be identified from GRO-seq data by dREG (Danko et al., 2015; Wang et al., 2019). In addition, GRO-seq reads have been shown to be highly accumulated around active enhancer regions (±1 kb) (Danko et al., 2015; Hu et al., 2017; Wang et al., 2019). Therefore, we designated the enhancers that fall within 1 kb of the GRO-seq peaks that were called by dREG as active enhancers (**Figure 1**). The lncRNAs that overlapped with the active enhancers were defined as elncRNAs (**Figure 1**), consistent with the method described in a previous study (Pefanis et al., 2015). As a result, 5.02% of the lncRNAs were defined as elncRNAs in the GM12878 cell line.

### Identification of Enhancer–Promoter Interaction Pairs

Hi-C reads and Hi-C interaction matrixes of the GM12878 cell line generated by Rao et al. were downloaded from the GEO repository under accession number GSE63525 (Rao et al., 2014). Using the chromatin interactions from Hi-C data, the frequency of all enhancer–promoter interactions in the GM12878 cell line was calculated. For example, for an enhancer–promoter interaction pair, the interaction frequency was represented by the count of reads that were located in both gene promoter regions and enhancer regions. To calculate statistical confidence estimates for the interaction pairs, we used the method, *fit-HiC*, as proposed by Duan et al. (2010). Only enhancer–promoter interaction pairs with FDR <0.001 were retained.

We designated pairs that consisted of elncRNA-associated enhancers and their target promoters as elncRNA-associated enhancer–promoter interaction pairs. Other enhancer–promoter interaction pairs were defined as non-elncRNA pairs.

### Selection of Structuring Factors

Previous studies have proven that some specific elncRNAs regulate the expression of their target genes by recruiting TFs to the promoter regions of the target genes (Schaukowitch et al., 2014; Miao et al., 2018; Tsai et al., 2018). To find the links between the TFBSs on enhancer–promoter pairs and elncRNAs, we selected eight proteins that potentially influenced enhancer–promoter interactions. CTCF, RAD21, and SMC3 have been well studied in terms of their ability to influence chromatin structure (Rao et al., 2014; Hong and Kim, 2017). RNA POLII can arrange spatial organization and mediate some loop structures which are smaller than CTCF loops (Tang et al., 2015). Weintraub et al. found that YY1 is a structural regulator of enhancer–promoter interactions and facilitates gene expression (Weintraub et al., 2017). In addition to these well-studied structuring factors, we also used ReMap, an integrative ChIP-seq analysis of regulatory regions, to find candidate proteins that can potentially mediate chromatin interaction. ReMap was used to annotate all of the filtered chromatin interactions (FDR < 0.001), and the most enriched TFs in intersection (*p *< 1 × 10^-500^) were selected as candidate proteins (Cheneby et al., 2018). After excluding TFs that were not available in a public database or not expressed in the GM12878 cell line, we selected HDGF, GATAD2B and GABPA from the most enriched TFs as structuring factors. Previous study suggested that G-quadruplexes, stable four-stranded non-canonical DNA structures, potentially facilitate enhancer–promoter interactions (Hegyi, 2015; Hou et al., 2019). Therefore, we also selected G-quadruplex sequences, which were derived from the work of Chambers et al. (2015) and can form G-quadruplexes *in vitro*, as a structuring factor.

Although we have selected many structuring factors, a large amount of chromatin interactions are mediated by other TFs. Therefore, we used the ENCODE ChIP-seq data for 137 TFs in the GM12878 cell line, which were merged by ReMap, as an integrated factor. All of the raw data of the structuring factors are shown in **Table 1**.

**Table 1:** The structuring factor data analyzed in this study.

Structuring factors	GEO number	Reference
CTCF	GSM935611	(Consortium 2012, Pope et al. 2014)
RAD21	GSM935332	(Consortium 2012, Pope et al. 2014)
SMC3	GSM935376	(Consortium 2012, Pope et al. 2014)
RNA POLII	GSM803355	(Consortium 2012, Pope et al. 2014, Gertz et al. 2013)
YY1	GSM803406	(Consortium 2012, Pope et al. 2014)
HDGF	GSE91531	(Consortium 2012)
GATAD2B	GSE105881	(Consortium 2012)
GABPA	GSE96120	(Consortium 2012)
G-quadruplex sequence	GSE63874	(Chambers et al. 2015)
ReMap TFs		(Cheneby et al. 2018)

### ChIP-seq Data Analysis

All of the ChIP-seq data were generated by the ENCODE Consortium (Consortium, 2012) and can be retrieved from the GEO database using their accession number (**Table 1**). To identify ChIP-seq peak regions, we performed peak calling using MACS with the default parameters (Zhang et al., 2008).

### Normalized ChIP-seq Peak Values on Enhancer–Promoter Pairs

We mapped all selected structuring factors (**Table 1**) onto the identified enhancer–promoter pairs. We defined enhancers/promoters as being associated with a specific structuring factor if they overlapped with a peak region of the selected structuring factor data. For G-quadruplex sequences, the G4-seq values provided by Chambers et al. (2015) were used to characterize the signal values of G-quadruplexes on enhancers/promoters. The peak counts on enhancers/promoters were used to define the signal values of the merged TFs on enhancers/promoters. For other structuring factors, the peak values, which were calculated by MACS (Zhang et al., 2008), were used to define the signal values of the structuring factor of these enhancers/promoters. If multiple peaks of the certain structuring factor overlapped with one enhancer/promoter, the signal value of the structuring factor of the enhancer/promoter equals the maximum peak value.

Because most enhancer–promoter pairs are associated with several structuring factors and the ChIP-seq data of different structuring factors were from different experiments, the signal values on each enhancer–promoter pair were normalized. We used Z-score normalization to standardize different structuring factor signal values of enhancers/promoters.

$$Z_{ij}=\frac{x_{ij}-\mu_{i}}{\delta_{i}}\;(i\text{​​ }\in X;\;j\in Y)$$

Here, *Z* *_ij_* is the normalized signal value of the specified structuring factors (*i*) on a specified enhancer/promoter (*j*); the specified structuring factor (*i*) belongs to the structuring factors (*X*) in **Table 1**; the specified enhancer/promoter (*j*) belongs to previously identified enhancer–promoter pairs (*Y*); *x* *_ij_* represents the raw signal value of the specified structuring factors (*i*) on specified enhancer/promoter (*j*); *µ* *_i_* equals the average signal value of the specified structuring factor (*i*) of all enhancers/promoters (*Y*); and *δ* *_i_* indicates the standard deviation of the specified structuring factor (*i*) in all enhancers/promoters (*Y*).

### Clustering Enhancer–Promoter Pairs

We performed hierarchical clustering on elncRNA-associated enhancer–promoter pairs and other enhancer–promoter pairs in accordance with their normalized structuring factor signal values. The Clustering software (https://web.stanford.edu/group/sherlocklab/cluster.html) was used to cluster interaction pairs. The Pearson correlation was set as the distance measurement as described previously (Lan et al., 2012). Using all of the normalized signal values, the elncRNA-associated enhancer–promoter pairs and other enhancer–promoter pairs were clustered into 10 and 6 groups, respectively.

## Results

### ElncRNAs Are Highly Enriched in Chromatin Loop Anchors

**Figure 2:**
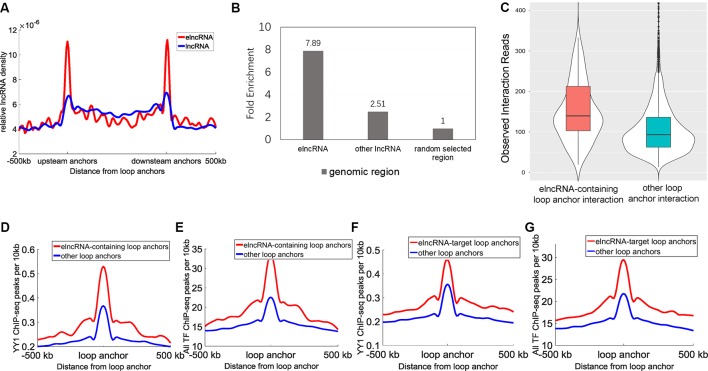
Relationship between elncRNAs and chromatin loops in the GM12878 cell line. **(A)** The distribution of elncRNAs and other lncRNAs across chromatin loops. The y-axis indicates the relative density of lncRNAs. Relative density was calculated from the ratio of the lncRNA counts per 10 kb to the total number of lncRNAs. The red line and blue line indicate elncRNA and other lncRNAs, respectively. **(B)** Enrichment of loop anchors with elncRNAs and other lncRNAs in the GM12878 cell line. The fold-enrichment was calculated by comparing the average counts of loop anchors overlapping per lncRNA to the average counts of loop anchors overlapping per random selected region. **(C)** The boxplot of Hi-C interaction reads between loop anchors. **(D**–**G)** The distribution of YY1 and the merged TF ChIP-seq peak counts surrounding loop anchors. The red lines and blue lines indicate elncRNA-containing loop anchors and other loop anchors, respectively. The distribution of YY1 **(D)** and the merged TF **(E)** ChIP-seq peak counts surrounding loop anchors. **(F**–**G)** The distribution of YY1 and the merged TF **(G)** ChIP-seq peak counts surrounding loop anchors.

A total of 9,449 high-confidence chromatin loops were identified in the GM12878 cell line. Each loop consisted of two interacting anchor points, which were defined as chromatin loop anchors. We calculated the relative density of elncRNAs and other lncRNAs across the entire chromatin loops (**Figure 2A**). We observed high accumulation of both elncRNAs and other lncRNAs at chromatin loop anchors, with the profiles found to gradually decline towards the central regions of chromatin loops (Student’s *t*-test, *p *= 1.08 × 10^-203^ and 5.77 × 10^-133^, respectively). Furthermore, the relative density of elncRNAs surrounding chromatin loop anchors was significantly higher than that of other lncRNAs (Student’s *t*-test, *p *= 3.16 × 10^-197^). The relative density of elncRNAs in the central regions of chromatin loops was slightly but significantly lower than that of other lncRNAs (Student’s *t*-test, *p *= 9.42 × 10^-27^). We next calculated the enrichment of loop anchors with elncRNAs (**Figure 2B**). The high enrichment of loop anchors with elncRNAs indicated that loop anchors tend to localize at sites where elncRNAs are produced, suggesting a potential role of elncRNAs in chromatin loops. Consistent with our observations, it has been reported that AS1eRNA, which is produced by the enhancer downstream of DHRS4-AS1, is involved in the formation of a loop between DHRS4-AS1 and its enhancer (Yang et al., 2016b). In this case, the enhancer and DHRS4-AS1 function as the loop anchors.

Moreover, the chromatin loop anchors containing elncRNAs displayed significantly higher loop anchor interactions (**Figure 2C**, Student’s *t*-test, *p *= 1.47 × 10^-22^), suggesting that elncRNAs at loop anchors potentially reinforce the interactions of loop anchors, which may help to maintain chromatin loop structures. The distribution of the architectural proteins including CTCF, SMC3, and RAD21 around loop anchors is shown in **Supplementary Figure 1**. Surprisingly, the ChIP-seq peak counts of these architectural proteins showed no significant differences between elncRNA-containing loop anchors and other loop anchors (**Supplementary Figures 1A–C**, Student’s *t*-test, *p *> 0.001), indicating that the high interaction strength of elncRNA-containing loop anchors does not arise from these architectural proteins. We found that YY1 ChIP-seq peak counts around elncRNA-containing loop anchors were significantly higher than those around other loop anchors (**Figure 2D**, Student’s *t*-test, *p* = 1.62 × 10^-27^). Using CLIP-seq, YY1 was found to be capable of interacting with nascent enhancer RNA at the active enhancer regions where it is bound to DNA (Sigova et al., 2015). In addition, YY1 was shown to promote DNA interactions and chromatin looping (Weintraub et al., 2017). These findings suggested that elncRNAs on loop anchors can function to ”trap” YY1, thereby increasing the strength of interaction between loop anchors (**Figures 2C, D**). We used ReMap to merge ChIP-seq data of 137 TFs in the GM12878 cell line (Cheneby et al., 2018). The distribution of these TF ChIP-seq peaks around loop anchors is shown in **Figure 2E**. Likewise, we found that the merged TF ChIP-seq peak counts around elncRNA-containing loop anchors were significantly higher than those around other loops (**Figure 2E**, Student’s *t*-test, *p *= 1.94 × 10^-71^). These results suggested that the highly abundant TFBSs on elncRNA-containing loop anchors promoted the transcription of elncRNAs. As feedback regulatory elements, elncRNAs on loop anchors can facilitate the loop anchor interactions by recruiting TFs such as YY1.

We used Hi-C interaction pairs to select the loop anchors that interact with elncRNA genes (FDR <0.001); these anchors were defined as elncRNA-target loop anchors. Interestingly, the elncRNA-target loop anchors were also rich in YY1 ChIP-seq peaks and the merged TF ChIP-seq peaks (**Figures 2F, G**, Student’s *t*-test, *p *= 2.57 × 10^-24^ and 2.59 × 10^-63^ for YY1 and all TF ChIP-seq, respectively). These results suggested that elncRNAs not only influenced loop anchors locally but also potentially affected the target loop anchors through higher-order chromatin structures.

### ElncRNAs Are Associated With the Interactions Between Enhancers and Promoters

**Figure 3:**
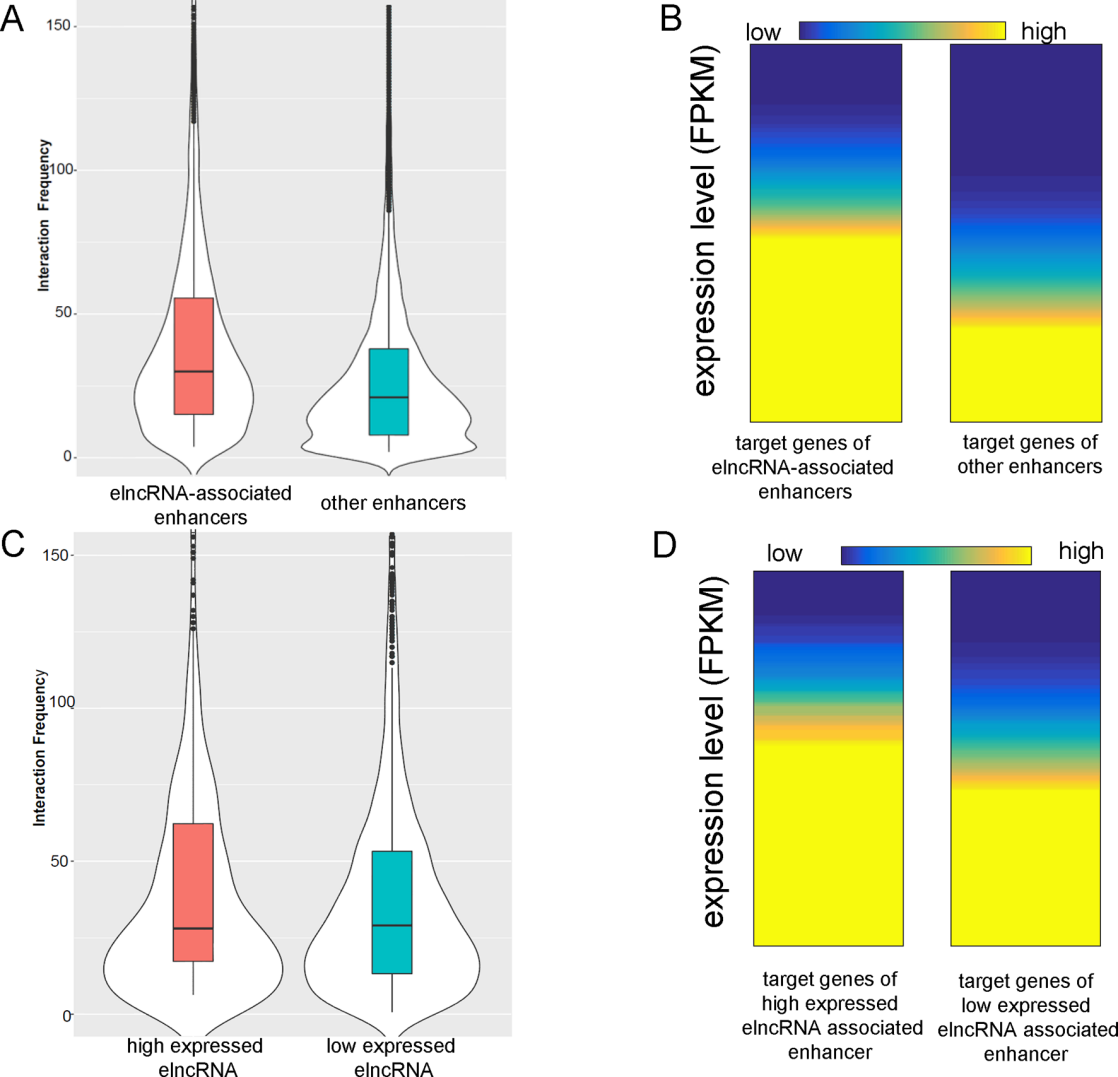
ElncRNAs are associated with enhancer–promoter interactions. **(A)** Interaction frequency of elncRNA-associated enhancer–promoter pairs and other enhancer–promoter pairs. **(B)** Expression levels of target genes of different enhancers. **(C)** Interaction frequency of differently expressed elncRNA-associated enhancer–promoter interactions. **(D)** Expression levels of target genes of elncRNA-associated enhancers.

The average interaction frequency (49.32) of elncRNA-associated enhancer–promoter pairs was significantly higher than that (39.28) of other enhancer–promoter pairs (**Figure 3A**, Student’s *t*-test, *p* = 1.11 × 10^-33^). Moreover, the expression levels of the target genes of elncRNA-associated enhancers (average FPKM = 58.97) were significantly higher than those of other enhancers (**Figure 3B**, average FPKM = 26.61, Student’s *t*-test, *p* = 3.77 × 10^-27^), suggesting that the stable interactions of elncRNA-associated enhancer–promoter pairs lead to high expression levels of the target genes.

The elncRNAs were divided into two equal groups with high and low expression levels using the FPKM values; the 50% with the lower FPKM were defined as low expressed elncRNAs, and the 50% with the higher FPKM were defined as high expressed elncRNAs. The interaction frequency (52.88) of high expressed elncRNA-associated enhancer–promoter pairs was significantly higher than that of low expressed elncRNA-associated pairs (48.14, **Figure 3C**, Student’s *t*-test, *p* = 7.34 × 10^-17^). The expression levels of the target genes of high expressed elncRNA-associated enhancers (average FPKM = 64.47) were also significantly higher than those of low expressed elncRNA-associated enhancers (**Figure 3D**, average FPKM = 55.79, Student’s *t*-test, *p* = 4.19 × 10^-12^).

### ElncRNAs Are Involved in Enhancer–Promoter Interactions in Different Ways

**Figure 4:**
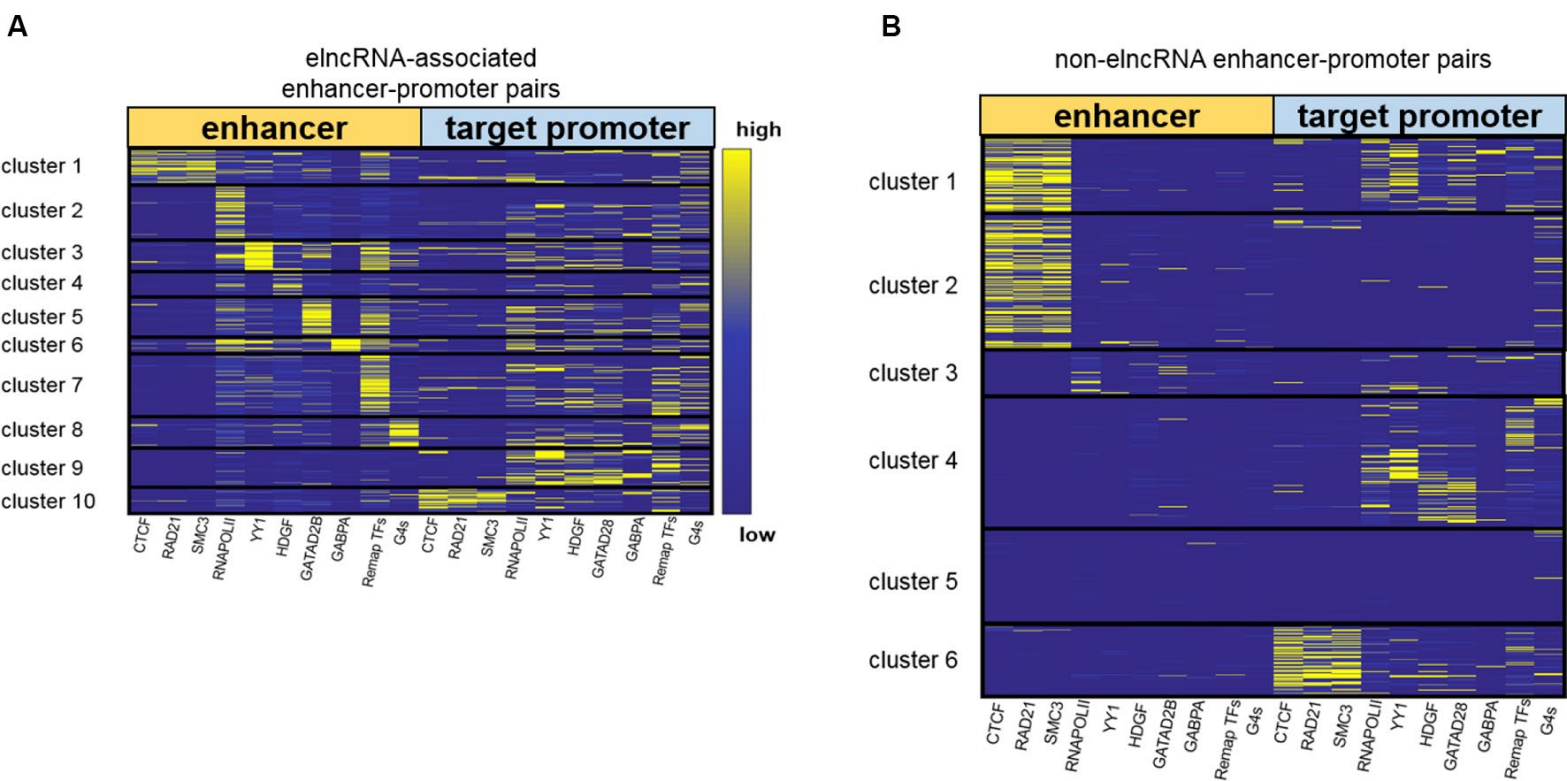
Clustering enhancer–promoter pairs. Heatmap of structuring factor signals on enhancer–promoter pairs. **(A)** ElncRNA-associated enhancer–promoter pairs were clustered into 10 groups using hierarchical clustering according to the various structuring factor signal values. **(B)** Non-elncRNA enhancer–promoter pairs were clustered into 6 groups using hierarchical clustering.

Using the signal values of structuring factors on enhancer–promoter pairs, we clustered elncRNA-associated enhancer–promoter pairs into 10 groups (**Figure 4A**). In comparison, non-elncRNA enhancer–promoter pairs can be clustered into 6 groups (**Figure 4B**). Although previous research proved that CTCF and cohesin proteins are involved in enhancer–promoter interactions (Li et al., 2015; Tang et al., 2015; Rao et al., 2017), we found that only a small proportion of elncRNA-associated enhancer–promoter interactions (cluster 1 and cluster 10, 20.99%) depended on these architectural proteins. In contrast, most non-elncRNA enhancer–promoter pairs (cluster 1, cluster 2 and cluster 6, 55.92%) were significantly rich in CTCF and cohesin proteins (**Figure 4**, Student’s *t-test*, *p* = 8.95 × 10^-146^, 1.21 × 10^-120^, and 1.94 × 10^-102^ for CTCF, RAD21, and SMC3, respectively).

To produce elncRNAs, it is reasonable that RNA POLII and YY1 were highly accumulated around elncRNA-associated enhancers. ElncRNA-associated enhancers in clusters 2 and 3 have much higher RNA POLII and YY1 signal values than other enhancers (**Figure 4A**), suggesting that these enhancer–promoter interaction pairs are highly related to RNA POLII and YY1 binding. Intriguingly, the enhancers of non-elncRNA pairs in cluster 3 (**Figure 4B**) also have some RNA POLII signal values, which may contribute to these non-elncRNA enhancer–promoter interactions.

We found that HDGF preferentially localizes at elncRNA-associated enhancers in cluster 4 (**Figure 4A**). HDGF is involved in protein–protein, protein–RNA, and protein–DNA interactions (Zhao et al., 2011; Bao et al., 2014). Our results suggested that elncRNA potentially attracts HDGF to local enhancers and HDGF facilitates enhancer–promoter interactions through protein–protein or protein–DNA interactions. GATAD2B binding sites were abundant on elncRNA-associated enhancers in cluster 5 (**Figure 4A**). Jing et al., (2008) proposed that GATA factors are tightly linked to the chromatin interactions. Our results showed that some enhancer–promoter interaction pairs were associated with GATA factors. GABPA binding sites tend to distribute around elncRNA-associated enhancers in cluster 6. In line with our observations, the binding of GABPA was reported to be capable of mediating long-range chromatin interactions and upregulating transcription (See et al., 2019).

In addition to the TFs discussed above, a large number of elncRNA-associated enhancer–promoter interaction pairs were influenced by other TFs. Compared with non-elncRNA enhancers, most elncRNA-associated enhancers contain enriched TF ChIP-seq peaks, especially in cluster 7. The enhancers and promoters in cluster 7 were brought together by the enriched TFs (**Figure 4A**). Additionally, G-quadruplex sequences were also associated with some enhancer–promoter interaction pairs (**Figure 4A**). In line with our findings, recent research suggested that G-quadruplexes on enhancers and promoters might facilitate enhancer–promoter interactions (Hegyi, 2015; Hou et al., 2019).

Together, these results show that elncRNAs regulate the enhancer–promoter interactions in different ways. Only a fraction (22.9%) of elncRNA enhancer–promoter pairs contained architectural protein binding sites including CTCF, SMC3 and RAD21. Most elncRNA enhancers contained RNA POLII, which can mediate chromatin interactions and is highly related to elncRNA transcription. YY1, HDGF, GATAD2B, and GABPA were also enriched in parts of elncRNA-associated enhancers. These structuring factors potentially facilitate some elncRNA-associated enhancer–promoter interactions. In addition to the TFs, G-quadruplex sequences, which were highly associated with chromatin structures, were found to be enriched in cluster 8 of elncRNA-associated pairs.

To investigate whether the cluster results were influenced by the number or the choice of structuring factors, we used different numbers of structuring factors to cluster enhancer–promoter interaction pairs (**Supplementary Figures 2A, B**). We retained CTCF, RAD21, SMC3, RNA POLII, the merged TFs, and G-quadruplex sequences as the 6 structuring factors. Using these factors, the elncRNA-associated enhancer-promoter pairs can be clustered into 6 groups (**Supplementary Figure 2A**). Because YY1, HDGF, GATAD2B, and GABPA were removed, the pairs in clusters 3–6 of **Figure 4A**, which had enhancers enriched in these TFs, were clustered into different groups according to their structuring factor signal values (**Supplementary Figure 2A**). However, 90.82% of the pairs in the other clusters of **Figure 4A** clustered back into the same groups, in which the enhancers were rich in the architectural protein, RNA POLII, the merged TFs, and G-quadruplex sequences, regardless of whether 6 or 10 structuring factors were used (**Figure 4A** and **Supplementary Figure 2A**). We further added six more structuring factors—NRF1, HSF1, NRSF, MAX, MAZ, and CHD1—to our structuring factor candidates (a total of 16 structuring factors). These TFs are known to be involved in the regulation of chromatin structure (Garriga-Canut et al., 2006; Smolle et al., 2012; Domcke et al., 2015; Sadeghifar et al., 2015; Zhang et al., 2016; Fujimoto et al., 2017). The elncRNA-associated enhancer-promoter pairs were clustered into 11 groups in accordance with the 16 structuring factor signals (**Supplementary Figure 2B**). We found that 82.22% of the elncRNA-associated enhancer-promoter pairs have the same clustering results regardless of whether 10 or 16 structuring factors were used (**Figure 4A** and **Supplementary Figure 2B**). The enhancers in cluster 11 of **Supplementary Figure 2B** were rich in the CHD1 ChIP-seq peaks, but only 1.59% of the elncRNA enhancer-promoter pairs were clustered into cluster 11. Furthermore, the signal values of NRF1, HSF1, NRSF, MAX, and MAZ on elncRNA-associated pairs were quite low and dispersed, indicating that these proteins were only marginally involved in the elncRNA-associated enhancer-promoter interaction pairs. Therefore, only the most commonly used structuring factors (the 10 structuring factors in **Table 1**) were retained.

**Figure 5:**
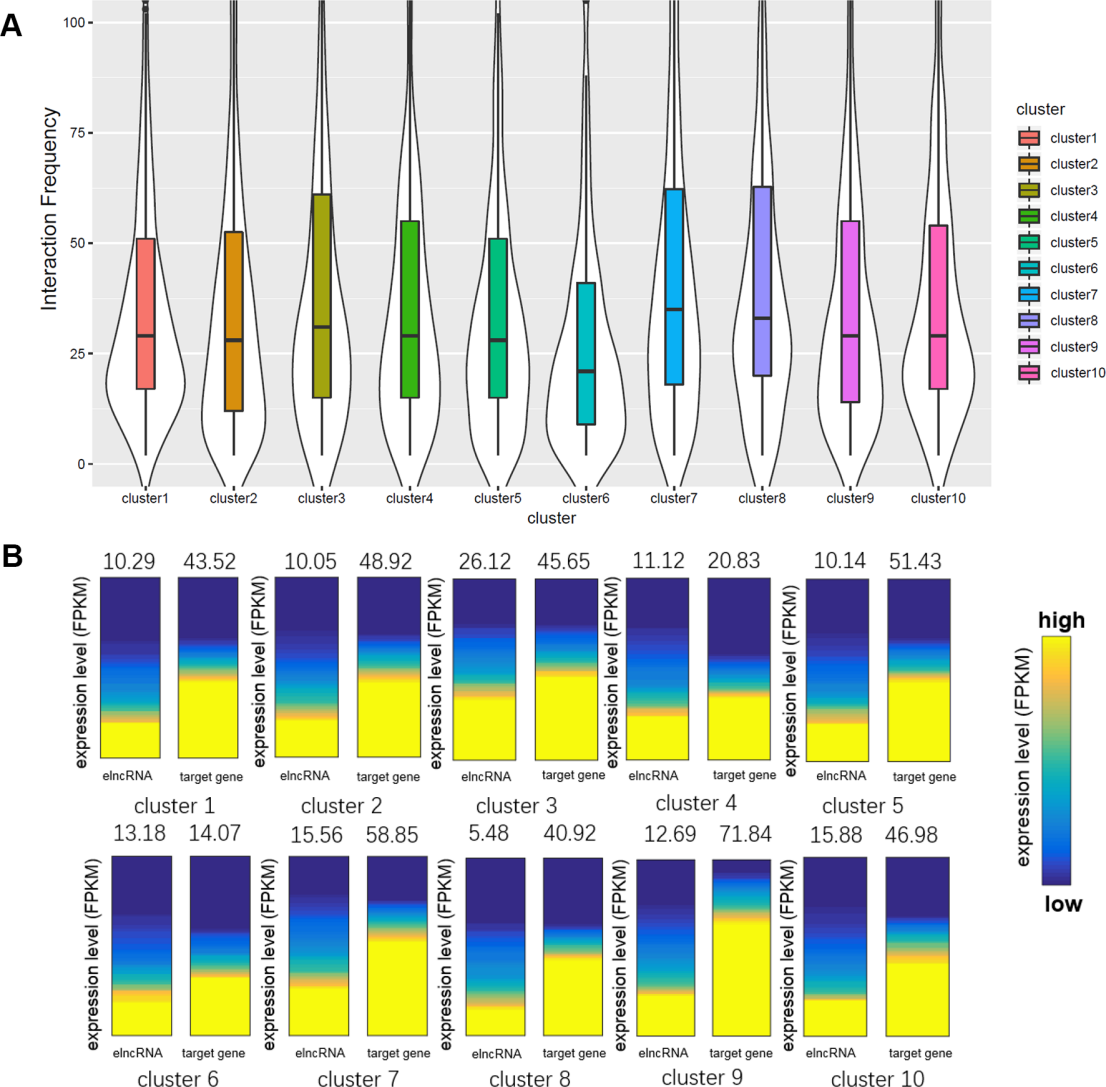
Comparison of the enhancer–promoter pairs in different clusters. **(A)** Boxplot of interaction frequency of different clusters. **(B)** Gene expression analyses of different clusters. The average expression levels were indicated above the heatmaps. The heatmaps represent the expression levels of elncRNAs and their target genes in different clusters. The genes were sorted according to their expression levels.

We calculated the interaction frequency of elncRNA-associated enhancers in the different clusters (**Figure 5A**). Interestingly, elncRNA-associated enhancer–promoter pairs in cluster 8, which were highly associated with G-quadruplex sequences, displayed the highest interaction frequency, suggesting that the enhancer–promoter pairs mediated by G-quadruplexes were quite stable. In addition, elncRNA-associated enhancer–promoter pairs in cluster 7 (**Figure 5A**) also displayed significantly higher interaction frequency than other elncRNA-associated enhancer–promoter pairs (Student’s *t*-test, *p* = 2.56 × 10^-14^), suggesting a critical role of TFs in enhancer–promoter interactions. However, the elncRNA-associated enhancer–promoter pairs in cluster 6, which were rich in HDGF binding sites, displayed significantly lower interaction frequency (Student’s *t*-test, *p* = 4.97 × 10^-8^) than other elncRNA-associated enhancer–promoter pairs. It has been reported that the N-terminal PWWP domain of HDGF is required for DNA binding (Yang and Everett, 2007), but PWWP-DNA interactions could be weak and/or unstable (Morchikh et al., 2013). We suspected that the low interaction frequency of enhancer–promoter pairs mediated by HDGF may be explained by the unstable binding of HDGF.

Even though elncRNA-associated enhancer–promoter interaction frequency (cluster 8) was the highest, the elncRNAs in cluster 8 were expressed significantly lower than other elncRNAs (**Figure 5B**, Student’s *t*-test, *p* = 9.77 × 10^-44^). We suspected that the formation of G-quadruplexes in this cluster serve as a compensation for the low expressed elncRNAs. And the elncRNA-associated enhancer-target genes in cluster 4 and cluster 6 express significantly lower than other enhancer-target genes (Student’s *t*-test, *p *= 4.49 × 10^-22^ and 1.90 × 10^-58^ for cluster 4 and cluster 6, respectively), because enhancer–promoter interaction pairs in cluster 4 and cluster 6 were mainly mediated by HDGF and GABPA. HDGF has been reported to function as a transcriptional repressor (Yang and Everett, 2007), suggesting that elncRNAs promote HDGF binding on enhancers which further influence the expression of these enhancer-target genes. GABPA was found to be overrepresented in methylated regions (Hogart et al., 2012). We hypothesized that these interactions mediated by GABPA may be influenced by DNA methylation, which leads to the low expression of these target genes in cluster 6.

**Figure 6:**
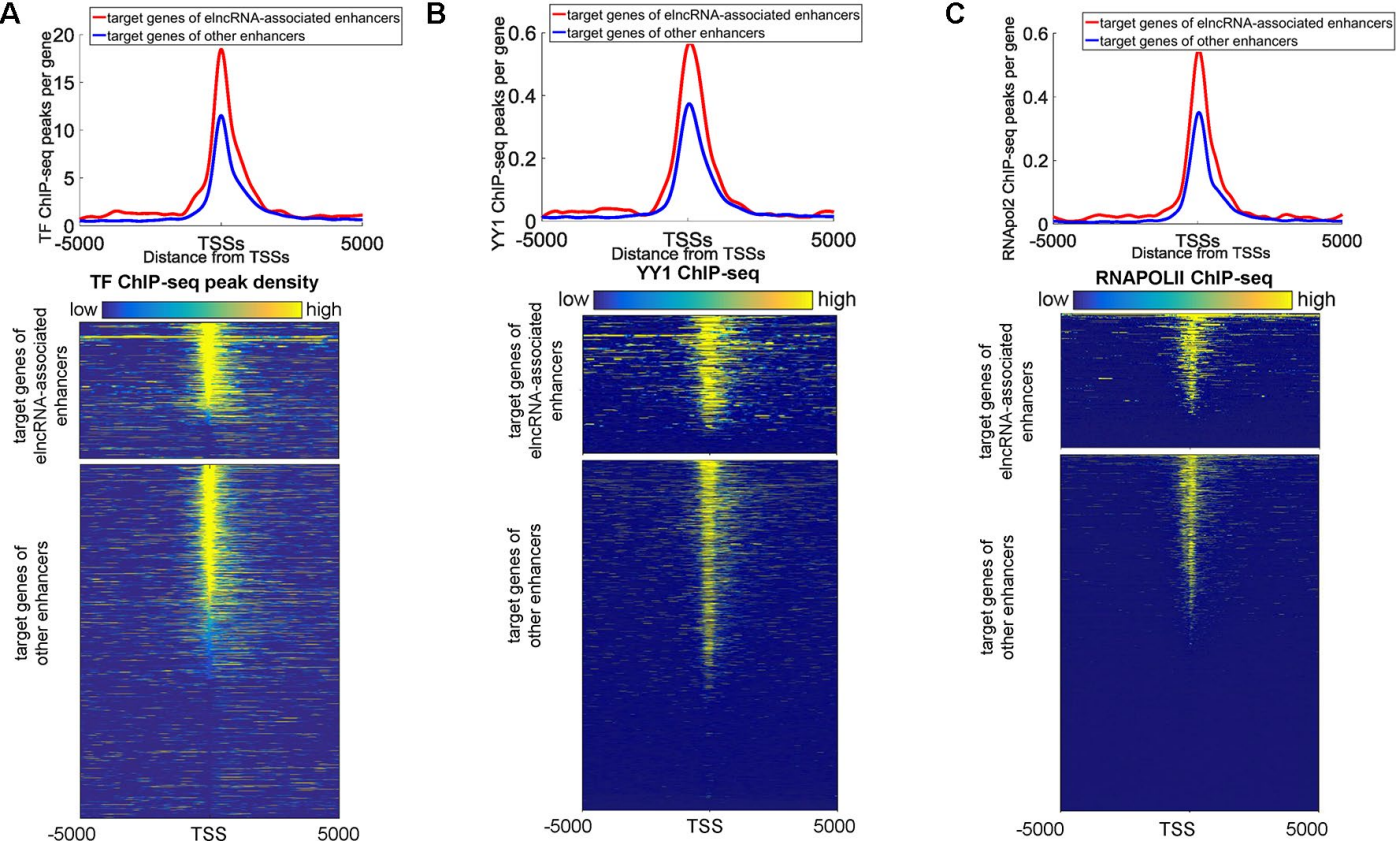
Distribution of ChIP-seq peaks around target genes of elncRNA-associated enhancers and other enhancers, respectively. The red lines and blue lines indicate target genes of elncRNA-associated enhancers and other enhancers, respectively. **(A)** Top panel: the distribution of ChIP-seq peak counts around target genes of elncRNA-associated enhancers and other enhancers. Bottom panel: Heatmap of all TF ChIP-seq peaks around TSSs; each row represents a target gene of enhancers. **(B)** Top panel: Distribution of YY1 ChIP-seq peak counts around target genes of elncRNA-associated enhancers and other enhancers. Bottom panel: Heatmap of YY1 ChIP-seq reads around TSSs. **(C)** Top panel: the distribution of RNA POLII peak counts around target genes of elncRNA-associated enhancers and other enhancers. Bottom panel: Heatmap of RNA POLII ChIP-seq reads around TSSs.

We also found that some protein binding sites displayed a strong bias towards the target promoters of elncRNA-associated enhancers, indicating that elncRNA can potentially influence target genes (in *trans*). We showed the ChIP-seq peaks of all merged TFs, YY1, and RNA POLII around the target genes of enhancers in the GM12878 cell line, respectively (**Figures 6A–C**). Compared with the target genes of other enhancers, the target genes of elncRNA-associated enhancers were significantly rich in TFBSs, especially for YY1 and RNA POLII (**Figures 6A–C**, Student’s *t*-test, *p* = 1.01 × 10^-36^, 1.03 × 10^-27^, and 4.42 × 10^-29^ for all TFs, YY1, and RNA POLII, respectively), suggesting that elncRNAs can influence some proteins, especially for YY1 and RNA POLII, on the target promoters (in *trans*) (**Figures 6A–C**).

### G-Quadruplexes Are Associated With the Expression of elncRNAs

**Figure 7:**
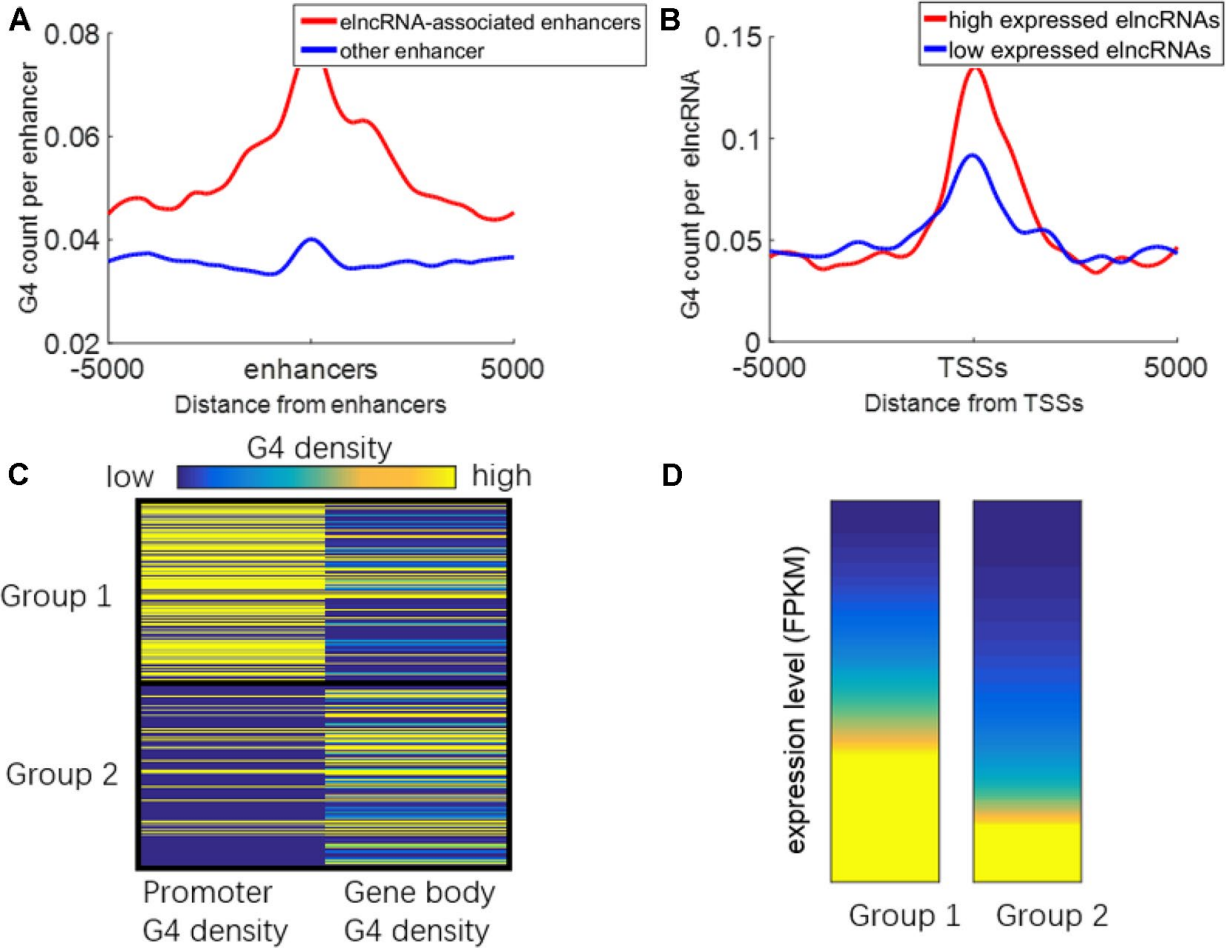
Relationship between G-quadruplexes and elncRNAs. **(A)** Distribution of G-quadruplex sequences around elncRNA-associated enhancers (red) and other enhancers (blue). **(B)** Distribution of G-quadruplex sequence around TSSs of high expressed elncRNAs (red) and low expressed elncRNAs (blue). **(C)** ElncRNAs were clustered into two groups according to the G-quadruplex sequence density of promoters and gene bodies. **(D)** The expression levels of elncRNAs in different groups.

It has been reported that G-quadruplexes show hallmarks of dynamic epigenetic features in chromatin primarily found in regulatory, nucleosome-depleted regions and correlate with high expressed genes (Hansel-Hertsch et al., 2016). Because some enhancers can be transcribed to produce elncRNAs, G-quadruplexes on enhancers may also be related to the transcription of enhancers. We suspected that G-quadruplexes on enhancers can facilitate enhancer transcription events. The distribution of G-quadruplex sequences around the enhancers is shown in **Figure 7A**. G-quadruplex sequence counts around elncRNA-associated enhancers were significantly higher than those around other enhancers (Student’s *t*-test, *p* = 1.28 × 10^-70^). Moreover, G-quadruplex sequence counts around TSSs of high expressed elncRNAs were significantly higher than those of low expressed elncRNAs (**Figure 7B**, Student’s *t*-test, *p* = 4.33 × 10^-39^).

We clustered the elncRNAs into two groups (**Figure 7C**). In group 1, G-quadruplex sequences were preferentially localized in promoters of elncRNAs rather than gene body regions. In group 2, G-quadruplex sequences were more likely to be distributed along elncRNA gene body regions. The expression levels of elncRNAs in group 1 were significantly higher than those in group 2 (**Figure 7D**, Student’s *t*-test, *p* = 7.13 × 10^-29^). Because G-quadruplexes on the gene body may stall elongation of RNAPOLII, high enrichment of G-quadruplex sequences on gene bodies will lead to the low expressed level of elncRNAs. However, G-quadruplex sequences on promoters are highly related to chromatin accessibility, and G-quadruplexes can recruit transcription factors to promoters, which can further promote the expression levels of elncRNAs. We inferred that high abundant G-quadruplex sequences in elncRNA promoters facilitated the steady expression of elncRNA.

## Discussion

There is a broad consensus that enhancers can generate non-coding transcripts (Li et al., 2016). Nevertheless, whether these non-coding transcripts are functional or merely a byproduct remains poorly understood. Some studies proved that some specific enhancer RNAs play critical roles in biological processes (Lai et al., 2013; Li et al., 2013; Melo et al., 2013; Schaukowitch et al., 2014; Yang et al., 2016a; Tsai et al., 2018; Miao et al., 2018). However, deciphering the function and mechanism of elncRNAs in the genome-wide range remains a challenge. In this study, we characterized elncRNAs in human chromatin structures. Using both GRO-seq and RNA-seq data, we identified active enhancers and elncRNAs of the GM12878 cell line (**Figure 1**). ElncRNAs were significantly enriched in chromatin loop anchors (**Figure 2A**). It is well accepted that loop extrusion should depend on either cohesin slides or ATP-driven motors including transcription and DNA replication (Davidson et al., 2016; Busslinger et al., 2017; Ganji et al., 2018; Vian et al., 2018). We found that chromatin loop anchors are prone to being localized around genomic regions where elncRNAs are expressed (**Figures 2A, B**). Our findings suggested that the transcription of elncRNAs is involved in the formation of chromatin loop structures. Moreover, chromatin loops with anchors containing elncRNAs are more stable than those lacking elncRNAs (**Figure 2C**). The chromatin loop anchors always contain abundant architectural protein binding sites regardless of whether there are elncRNAs on them (**Supplementary Figure 1**). However, the YY1 and RNA POL II ChIP-seq signal values of elncRNA-containing loop anchors were significantly higher than those of other loop anchors. It has been reported that YY1 can be recruited by elncRNAs to active enhancer regions (Sigova et al., 2015) and YY1 can mediate chromatin interactions (Weintraub et al., 2017). Our results suggested that the high enrichment of RNA POLII and TFBSs, especially for YY1 binding sites, promotes the stable interactions between elncRNA-containing anchors (**Figures 2D–G**).

We also found that elncRNAs were potentially involved in maintaining enhancer–promoter interactions in the genome-wide range. The interaction frequency of elncRNA-associated enhancer–promoter pairs was significantly higher than that of other enhancer–promoter pairs (**Figure 3A**). Furthermore, the frequent enhancer–promoter interactions led to significantly higher expression levels of these genes (**Figure 3B**). Additionally, the interaction frequency (52.88) of high expressed elncRNA-associated enhancer–promoter pairs was significantly higher than that of low expressed elncRNA-associated pairs (48.14, **Figure 3C**, Student’s *t*-test, *p* = 7.34 × 10^-17^). The expression levels of genes interacting with high expressed elncRNA associated enhancers (average FPKM = 64.47) were also significantly higher than those of genes (average FPKM = 55.79) interacting with other enhancers (**Figure 3B**, Student’s *t*-test, *p* = 4.19 × 10^-12^). Our results suggested that the high expression levels of target genes of elncRNA-associated enhancers might arise from the high enrichment of TFBSs including YY1 on the target promoters (**Figures 6A, B**). As discussed above, YY1 can be recruited by elncRNAs and mediate enhancer–promoter interactions. We inferred that the close association between YY1 and elncRNAs can facilitate the interaction of elncRNA-associated enhancers and their target promoters.

Although our results showed that elncRNAs were highly associated with the high interaction frequency of enhancer-promoter pairs, it remains unclear whether all of these elncRNAs are functional. Because only a few elncRNAs have been proven to be functional with experimental support, further experimental research and more convincing evidence are still needed. In addition, whether elncRNAs have specific distinguishing features compared with other long non-coding transcripts needs further investigation. The causal relationship between enhancer transcripts and enhancer-promoter interactions also requires further study.

To further evaluate the role of elncRNAs in enhancer–promoter interactions, we clustered enhancer–promoter pairs into different groups based on the structuring factor signal values (**Figures 4A, B**). The enhancers in elncRNA-associated pairs contained abundant TFBSs. However, the enhancers in non-elncRNA pairs were primarily rich in CTCF and cohesin proteins. Although previous research proved that CTCF and cohesin proteins are important for enhancer–promoter interactions (Li et al., 2015; Tang et al., 2015; Rao et al., 2017), we found that only a small portion of elncRNA-associated enhancer–promoter interactions (cluster 1 and cluster 10, 20.99%) were rich in these architectural proteins (**Figure 4A**). In contrast, more than 55.92% non-elncRNA enhancer–promoter interaction pairs (cluster 1, cluster 2, and cluster 6) are rich in the architectural protein binding sites (**Figures 4A, B**, Student’s *t*-test, *p* = 8.95 × 10^-146^, 1.21 × 10^-120^, and 1.94 × 10^-102^ for CTCF, RAD21, and SMC3, respectively). It was found that elncRNA-associated enhancers in cluster 2 have much higher RNA POLII signal values than other enhancers (**Figure 4A**). Interestingly, the enhancers in cluster 3 of non-elncRNA pairs also contained RNA POLII. We hypothesized that the RNA POLII on the non-elncRNA enhancers was caused by frequent interaction between active genes and these enhancers. Unlike elncRNA-associated pairs, a part of non-elncRNA pairs have almost no structuring factor signal values (cluster 5 in non-elncRNA pairs). In this context, it is possible that these pairs are mainly located in heterochromatin, leading to the lack of TF binding. Compared with other enhancers, elncRNA-associated enhancers contain various TFBSs, suggesting that elncRNAs are involved in enhancer–promoter interactions in different ways. Most elncRNA-associated enhancers contained abundant TF ChIP-seq peaks, which can promote the activity of enhancers and facilitate enhancer–promoter interactions. RNA POLII and YY1, which are able to mediate chromatin interactions and are highly related to elncRNA transcription, were enriched in parts of elncRNA-associated enhancers. HDGF, GATAD2B, and GABPA also potentially facilitate some enhancer–promoter interactions. In addition, these proteins have been proven to be associated with chromatin interactions by protein-protein interactions or DNA-protein interactions (Jing et al., 2008; Hogart et al., 2012; Bao et al., 2014). G-quadruplexes, the non-canonical secondary structures formed in guanine-rich nucleic acid sequences, are highly associated with gene regulation. We found that G-quadruplex sequences were enriched in cluster 8 of elncRNA-associated pairs. In addition to the identified differences, we also found some similarities. For example, we found that both elncRNA-associated pairs and non-elncRNA pairs include some interaction pairs consisting of the enhancers that lack all of the structuring factors (cluster 9 in elncRNA-associated pairs and cluster 4 in non-elncRNA pairs). However, the target promoters in these pairs contain abundant TFBSs, such as YY1, HDGF, and GATAD2B. The interactions of these pairs may be facilitated by the enriched TFs on the promoters. Our study mainly revealed the association between elncRNAs and the enrichment of TFs on elncRNA-associated pairs. Even though it has been widely accepted that lncRNAs can attract proteins by their specific secondary structure, the causal relationship between elncRNAs and TFs still requires further experimental validation. In addition, the internal mechanism by which different elncRNAs attract different TFs remains unknown.

G-quadruplex sequences, which can form G-quadruplexes in vitro, were significantly accumulated around elncRNA-associated enhancers (**Figure 7A**). Moreover, the levels of G-quadruplex sequences at elncRNA-associated enhancers were significantly higher than other enhancers (**Figure 7A**). The formation of G-quadruplex structures can stabilize the R-loop structures consisting of the nascent RNA and unwound template DNA (Skalska et al., 2017). The high enrichment of G-quadruplexes on elncRNA-associated enhancers is capable of promoting the stability of R-loop structures consisting of elncRNAs and their template DNA. The retained elncRNAs can potentially influence target promoters through enhancer-promoter interactions. Furthermore, G-quadruplex sequence counts around TSSs of high expressed elncRNAs were also significantly higher than those of low expressed elncRNAs (**Figure 7B**). Because G-quadruplexes in promoters are highly associated with elevated transcriptional genes, we hypothesized that high expressed elncRNAs are related to the enrichment of G-quadruplex sequences in their promoters. Although abundant G-quadruplex sequences in elncRNA promoters may be related to the steady expression of elncRNAs, G-quadruplex sequences in gene bodies of elncRNAs may prevent the expression of elncRNAs by stalling the elongation of RNA POLII (**Figures 7C, D**).

## Data Availability Statement

Publicly available datasets were analyzed in this study. This data can be found here: GSE60456, GSE90223, GSE63525, GSM935611, GSM935332, GSM935376, GSM803355, GSM803406, GSE91531, GSE105881, GSE63874, GSE96120.

## Author Contributions

Conceptualization: YH and XS. Methodology and experimentation: YH, RZ, and XS. Writing: YH and XS.

## Funding

This work was supported by the National Natural Science Foundation of China (61972084, 81830053) and the Key Research and Development Program of Jiangsu Province (BE2016002-3).

## Conflict of Interest

The authors declare that the research was conducted in the absence of any commercial or financial relationships that could be construed as a potential conflict of interest.
