# Supplementary material for: Enhancer LncRNAs Influence Chromatin Interactions in Different Ways
Source: Front Genet. 2019 Oct 16;10:936. doi: 10.3389/fgene.2019.00936 (PMC6807612; doi:10.3389/fgene.2019.00936)
Supplement: Supplementary file 1 [file Presentation_1.pdf]

Supplementary Material

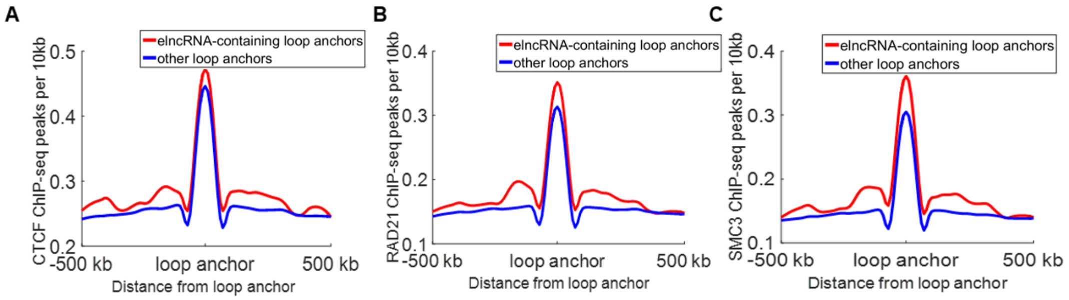

Supplementary Figure 1. The architectural protein ChIP-seq peaks around loop anchors. CTCF (A), RAD21 (B) and SMC3 (C) ChIP-seq peak counts per 10 kb per loop anchor.

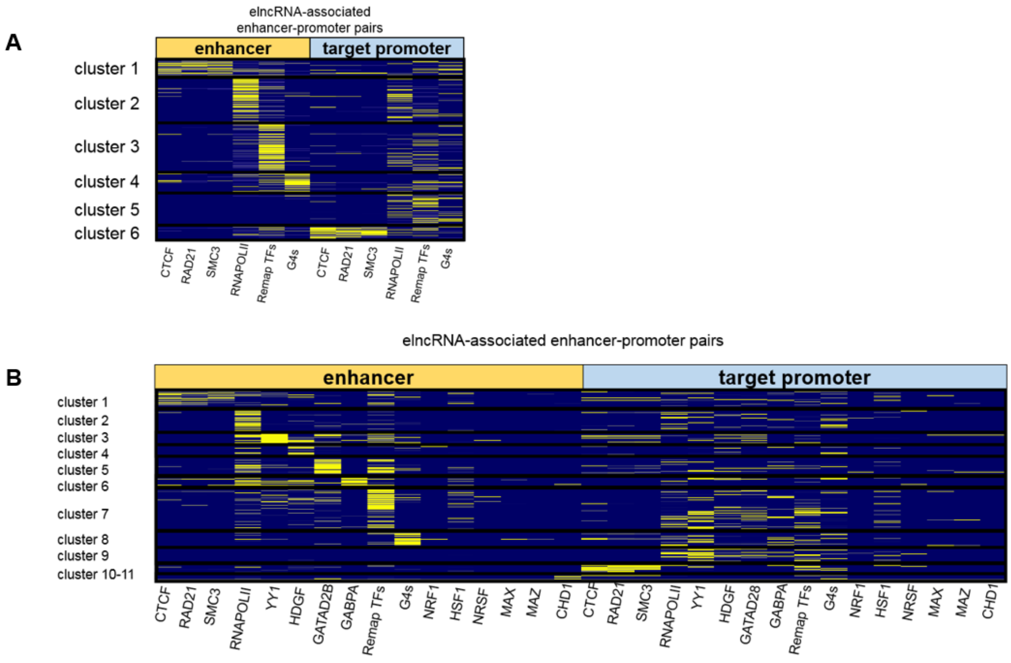

Supplementary Figure 2. Clustering enhancer-promoter pairs. (A) ElncRNA-associated enhancer–promoter pairs were clustered into 6 groups using hierarchical clustering in accordance with the signal values of the 6 structuring factors. (B) ElncRNA-associated enhancer–promoter pairs were clustered into 11 groups in accordance with the signal values of the 16 structuring factors.
